# Medical Education Blog and Podcast Utilization During the COVID-19 Pandemic

**DOI:** 10.7759/cureus.23361

**Published:** 2022-03-21

**Authors:** Patrick E Boreskie, Teresa M Chan, Chris Novak, Adam Johnson, Jed Wolpaw, Andrew Ong, Katherine Priddis, Pranai Buddhdev, Jessica Adkins, Jason A Silverman, Tessa Davis, James E Siegler

**Affiliations:** 1 Emergency Medicine, University of Manitoba, Winnipeg, CAN; 2 Emergency Medicine, McMaster University, Hamilton, CAN; 3 Pediatrics, University of Calgary, Calgary, CAN; 4 Vascular and Endovascular Surgery, Presbyterian Weill Cornell College of Medicine, New York, USA; 5 Anesthesia and Critical Care Medicine, Johns Hopkins University School of Medicine, Baltimore, USA; 6 Gastroenterology and Hepatology, Singapore General Hospital, Singapore, SGP; 7 Pediatric Emergency Medicine, Watford General Hospital, Watford, GBR; 8 Pediatric Orthopaedics, Broomfield Hospital, Broomfield, GBR; 9 Emergency Medicine, University of Kentucky, Lexington, USA; 10 Pediatric Gastroenterology and Nutrition, University of Alberta, Edmonton, CAN; 11 Pediatric Emergency Medicine, Royal London Hospital, London, GBR; 12 Neurology, Cooper University Hospital, Camden, USA

**Keywords:** blog, podcast, medical education, covid-19, online

## Abstract

Introduction

The coronavirus disease 2019 (COVID-19) pandemic disrupted traditional in-person learning models. Free Open Access Medical (FOAM) education resources naturally filled this void, so we evaluated how medical blog and podcast utilization changed during the early months of the pandemic.

Methods

Academic medical podcast and blog producers were surveyed on blog and podcast utilization immediately before (January-March 2020) and after (April-May 2020) the COVID-19 pandemic declaration and subsequent lockdown. Utilization is quantified in terms of blog post pageviews and podcast downloads. Linear regression was used to estimate the effect of publication during the COVID-19 period on 30-day downloads or pageviews. A linear mixed model was developed to confirm this relationship after adjustment for independent predictors of higher 30-day downloads or pageviews, using the podcast or blog as a random intercept.

Results

Compared to the pre-pandemic period, downloads and pageviews per unique blog and podcast publication significantly increased for blogs (median 30-day pageviews 802 to 1860, p<0.0001) but not for podcasts (median 30-day downloads 2726 to 1781, p=0.27). Publications that contained COVID-19 content were strongly associated with higher monthly utilization (β=7.21, 95% CI 6.29-8.14 p<0.001), and even non-COVID-19 material had higher utilization in the early pandemic (median 30-day downloads/pageviews 868 to 1380, p<0.0001).

Discussion

The increased blog pageviews during the early months of the COVID-19 pandemic demonstrated the important role of blogs in rapid knowledge translation. Podcasts did not experience a similar increase in utilization.

## Introduction

In our modern culture of instant access to online knowledge, medical education has had a surge in the use of asynchronous open educational resources (OERs) [1-5]. Online didactics, multimedia technology, and the ability to share content through social media have revolutionized learning and are of unique interest during the professional and educational disruption of the coronavirus disease 2019 (COVID-19) pandemic [6].

In the medical community, OERs used for knowledge dissemination are referred to as Free Open Access Medical education (FOAM). FOAM is an example of asynchronous education: the use of online resources, including videos, podcasts, and websites accessed by learners on their own time, and at a pace that matches learner preferences [7]. There is an increasing body of evidence that suggests these newer modalities are preferred by learners and are as effective, if not more so, for knowledge acquisition, than traditional forms of learning [8]. Two of the most popular forms of FOAM are blogs and podcasts, both of which have seen enormous growth over the past decade and are the focus of this study [2].

Prior to the pandemic, medical institutions tended to follow a traditional didactic model where learners relied on face-to-face gatherings like conferences and workshops and may have supplemented this content with occasional FOAM resources like blog posts or procedure tutorial videos [7]. With the spread of the novel coronavirus and the increasing lockdown measures worldwide, educators sought safer teaching strategies online. The FOAM community was poised to fill this need, having produced over a decade’s worth of multimedia didactics and tutorials, spanning a comprehensive curriculum of medical education. Though live video conferences were an option for continuing lectures and interactive discussions, asynchronous FOAM may have taken a more central role, with learners requiring more support from FOAM resources than ever before. As blog and podcast producers, many members of this authorship group noticed an uptick in website visits and downloads after the COVID-19 lockdowns. As the pandemic unfolded, the FOAM community had the experience in social media to dexterously disseminate new information related to COVID-19 testing, treatment, and public health strategies through engaging and shareable content. Some institutional sources like governmental health organizations and medical journals, on the other hand, had to quickly adapt their traditionally slower periodical submission and review process to stay relevant and react to constantly changing information.

Given their broad scope, capacity for rapid dissemination, and the disruption of traditional education models, we hypothesized that blogs and podcasts experienced greater utilization during the early coronavirus pandemic. In this study, we analyzed how the use of these types of FOAM changed during the onset of the pandemic, which could have important implications for peer review, academic advancement, and the integration of asynchronous education when traditional models cannot be implemented.

## Materials and methods

Definitions

Throughout the manuscript, we use the term “Program” to refer to an audio or visual podcast that has produced a series of episodes or a blog that has published a series of online posts. Usage of these two types of programs is quantified in terms of “downloads” (the number of requests to download, livestream, or play a podcast episode) and “pageviews” (the number of requests to view a blog post webpage). Posted podcast episodes and blog posts are referred to inclusively as “publications.”

Study design

The ADVANCE (Advocates for Digital, Visual, Audio, and Networked Clinical Education) study was a post-pre survey of clinical FOAM producers regarding blog and podcast utilization before and during the first wave of the COVID-19 pandemic, with some focused analyses having been previously reported [9]. Participating producers were recruiting using a multimodal campaign similar to a previously established methodology that includes social media calls and direct outreach to individuals within a virtual community of practice [10]. Participants received no compensation for their work in ADVANCE. When approached about ethics approval, the Institutional Review Board of the data coordinating center (Cooper University Hospital) stated that the study did not meet the criteria for human subjects research, and approval was not required. Data will be made available to any qualified investigator upon reasonable emailed request.

Included programs

Programs were included if they were English-language clinical medicine podcasts or blogs (1) reporting greater than 2000 blog pageviews or podcast downloads per month on average during 2019, (2) affiliated with a clinical academic society or peer-reviewed medical journal, or (3) offering continuing medical education (CME) credit to their users. These characteristics were identified as indicators of programs with greater relevance and impact in medical education [11]. All programs were clinically focused with a target audience of health sciences students, medical graduate trainees, or practicing medical providers. Programs were excluded from the analysis if they had not published content prior to January 1, 2020, or could not provide data from the study period.

Program producers completed a web-based survey to determine their programs’ eligibility for inclusion in ADVANCE. Those meeting criteria for inclusion were then invited to self-report on further information regarding the characteristics of the program and detailed data on program engagement. The information requested included the target audience’s level of training, affiliation with an academic society or peer-reviewed medical journal, program downloads or pageviews, and provision of CME credit for program usage. Considering most website user data is not publicly available, data were collected via producer self-reporting. Participant producers were asked to provide individual download or pageview details of blog posts and podcast episodes, whether they included COVID-19 content, and the content theme for a given episode or blog post (for example, “case-based discussion”, “clinical review”, etc.). A Twitter account was used as an indicator of social media presence. Self-reported number of Twitter followers was collected as exploratory data aimed at determining the association between social media presence and program utilization. To protect each program’s anonymity, programs were grouped into categories based on median followers for reporting here.

Independent variable: barriers to traditional didactics

Usage of blogs and podcasts was compared between the immediate “pre-COVID-19 months,” defined as January-March 2020 based on the rapid lockdown of many countries in late March 2020 following the World Health Organization declaration of a global pandemic, and the early “COVID-19 months,” defined as April-May 2020. Data from three months were compared to two months as medians, as the study was conducted and concluded quickly during the early pandemic without funding or planned long-term follow-up.

Primary and secondary endpoints

The pre-specified primary endpoint of the analysis was the number of podcast episode downloads or total blog pageviews per month. This endpoint is consistent with recent literature in which downloads and pageview thresholds have served as markers of the impact of online medical resources for use in academic advancement [11]. Secondary endpoints included the number of downloads or pageviews for each episode or post within the first day, seven days, and 30 days after publication, as well as the number of podcast episodes and blog pages published in both time periods.

Statistical analysis

Multiple strategies were used to examine the differences in blog and podcast usage in the immediate pre-COVID-19 months and the early COVID-19 months. Categorical data were compared using the chi-square test or Fisher’s exact test when contingency table cell counts were 5 or less. The normality of continuous data was assessed histographically and confirmed using the Shapiro-Wilk test. Non-normally distributed continuous data were compared between each program (when stratified by podcast or blog) using the Kruskal-Wallis equality of populations rank test, with Dunn’s pairwise correction using the Holm-Sidák method for multiple testing as previously described [12]. An unpaired t-test was used to determine the difference in monthly content (podcast episodes or blog posts) created before the coronavirus pandemic and the monthly content created during the early pandemic. Pearson correlation coefficients were generated to estimate relationships between continuous variables.

The effect of the early COVID-19 pandemic on the number of page views or downloads was estimated using linear regression, with fixed-effect estimates provided as beta coefficients (β) with 95% confidence intervals, with estimates provided for every 1000 downloads or pageviews. Due to the non-normal distribution of download and pageview counts, these values were log-transformed in order to generate the linear regression models. A multilevel linear mixed model was built to determine statistically significant predictors of higher 30-day downloads and pageviews, including all variables significant to p<0.2 in unadjusted regression. This model was generated using an unstructured covariance with three levels, including random intercepts at both the Podcast-versus-Blog and at the Program-within-Podcast-versus-Blog levels. To analyze the impact of program and content specifics, differences in usage were also examined using the Wilcoxon rank-sum test, with prespecified subgroup analyses of 30-day downloads stratified according to program characteristics: provision of CME credit, affiliation with academic society or peer-reviewed journal, episode format (clinical review, narrative story, case-based discussion, interview with author, or other), presence of COVID-19 content, and target audience (trainees or other).

All analyses were conducted using STATA 15.0 (College Station, TX) on the two-sided level. P-values are provided for convention with a significance level set at 0.05. Beyond the groupwise comparisons for pageviews and downloads, which were adjusted as described previously, no adjustments were made for multiple hypothesis testing. This analysis was exploratory, and sample size calculations were not made prior to data collection due to unanticipated response rates from producers and unknown download/pageview counts for their respective programs.

## Results

Participant demographics

**Figure 1:**
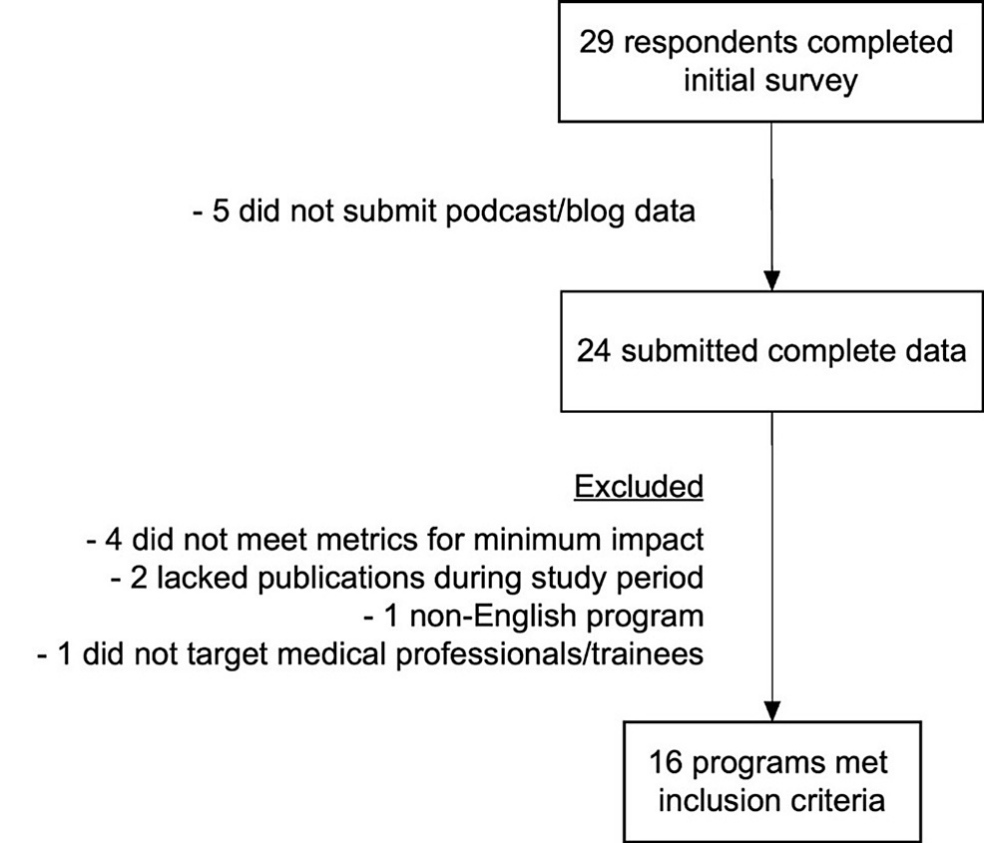
Inclusion flowchart

Of the 29 programs that responded to the initial survey, 24 submitted detailed engagement data on podcast episodes or blog posts, and 16 ultimately met inclusion criteria for analysis (Figure 1). Thirteen of the 16 programs featured at least one post or episode about COVID-19 (81%). Eight programs were affiliated with an academic society or peer-reviewed publication (50%), four offered CME credit (25%), and all had an affiliated Twitter account (median 10,700 followers (IQR 1695-21,200)). More than 95% of all published content targeted trainees (97.0%) or practicing medical professionals (99.5%), with <5% of content targeting non-medical listeners (2.8%) or patients (3.5%). Due to low event rates, subgroup analyses were not performed based on the target audience. 

Engagement before and during COVID-19

There were 198 podcast episodes published between January and May 2020 (103 in the pre-COVID-19 period and 95 in the COVID-19 period), with 650 unique blog posts (491 pre-COVID-19 and 159 during COVID-19), for a total of 848 unique publications during the whole study period. There were no significant differences between the number of posts or episodes across participating programs (median 20 (IQR 8-35), p_k-w_=0.46), although one outlier reported 671 posts (blog; 61.7% of total content). There was a strong correlation between the number of publications by a given program and the number of monthly downloads or pageviews per unique publication (r^2^=0.57, p<0.0001) but no correlation between the number of Twitter followers and monthly downloads (r^2^=0.007, p=0.40) or unique publications (r^2^=0.005, p=0.49).

Compared to the pre-pandemic months, the number of newly published blog posts or podcast episodes per month numerically decreased from a monthly mean of 198 to 127 (a 35.9% decline, p=0.25). Despite the decline in the number of new publications, there was a significant increase in the daily, weekly, and monthly pageviews or downloads of new content (Table 1). The considerable increase in downloads and pageviews of new program content during COVID-19 was driven predominantly by blogs, both because there was a greater number of blog posts than podcast episodes published and that there was a higher number of pageviews for each new blog post during COVID-19 (β=0.018, 95%CI 0.012-0.023, p<0.001). There was no significant difference in podcast episode downloads during the COVID-19 period versus the preceding months (p>0.05 for 24-hr, 7-day, and 30-day windows; Table 1). When engagement with new and old publications was combined, programs during COVID-19 had no difference in their total pageviews or downloads per month. Due to the unexpectedly high contributions of a single blog (accounting for 67.1% of all blog/podcast publications and 74.6% of all blog posts in this analysis), we performed post hoc comparisons of 30-day pageviews for this blog and for the remaining three blogs. Compared to 30-day pageviews before COVID-19, there were significantly higher monthly pageviews per post for this singular blog during COVID-19 (median 4145 (IQR 2157-9464) during COVID-19 vs. 903 (IQR 523-1538) pre-COVID-19, p<0.001). When this blog was removed from the analysis of the remaining blogs, there remained an increase in utilization when compared to prior months, however, this trend did not achieve statistical significance (median 537 (IQR 312-1071) during vs. 402 (IQR 267-802) pre-COVID-19, p=0.07).

**Table 1:** Primary analysis comparing pageviews/downloads in the pre-COVID-19 and early COVID-19 pandemic periods * Median downloads/pageviews per unique publication, with interquartile range † The number of publications with available data for a given cell are provided in parentheses. For example, of the total 594 publications released during the pre-COVID-19 period, data existed for all 594 regarding 30-day pageviews/downloads, but only 586 publications had data regarding 24-hour pageviews/downloads

	Pre-COVID-19 (Jan – Mar 2020) (N=594 publications)	COVID-19 (April – May 2020) (N=254 publications)	p-value
All unique publications (blog posts + podcast episodes)			
30-day pageviews/downloads	880 (437-1879)* (n=594)†	1817 (725-4231) (n=254)	<0.0001
7-day pageviews/downloads	473 (218-927) (n=590)	745 (354-2187) (n=251)	<0.0001
24-hour pageviews/downloads	171 (61-431) (n=586)	371 (136-812) (n=249)	<0.0001
Blog pageviews			
30-day pageviews	802 (397-1436) (n=491)	1860 (624-4897) (n=159)	<0.0001
7-day pageviews	435 (209-756) (n=491)	646 (331-1983) (n=159)	<0.0001
24-hour pageviews	133 (54-325) (n=491)	312 (124-712) (n=159)	<0.0001
Podcast downloads			
30-day downloads	2726 (791-3804) (n=103)	1781 (802-3667) (n=95)	0.27
7-day downloads	1484 (530-2575) (n=99)	1039 (523-2545) (n=92)	0.28
24-hour downloads	768 (284-1057) (n=95)	501 (174-1053) (n=90)	0.19

Subgroup analyses

Of the 848 unique episodes/posts that were published in the study period, 118 (13.9%) included COVID-19 content, 48 of which were published during the pre-COVID-19 period (40.7%). Compared to posts/episodes that did not feature COVID-19 content, those that featured COVID-19 content were associated with significantly higher 30-day downloads/pageviews (median downloads/pageviews 2892 (IQR 890-15487) vs. 935 (IQR 441-2167), p<0.001). Across study periods, there were significantly higher 30-day downloads/pageviews among non-COVID-19 content during the COVID-19 period (p<0.0001), but no significant difference in 30-day download/pageview counts among COVID-19 material during the COVID-19 period (p=0.10) when compared to the preceding months (Table 2). 

**Table 2:** Subgroup analyses evaluating 30-day pageviews/downloads in the pre-COVID-19 and early COVID-19 pandemic periods based on publication features * Median 30-day downloads/pageviews per unique publication (episode or blog post), with interquartile range † The number of publications with available data for a given cell are provided in parentheses. For example, of the total 594 publications released during the pre-COVID-19 period, 546 were not COVID-19 related while 48 were COVID-19 related.

	Pre-COVID-19 (Jan – Mar 2020) (N=594 publications)	COVID-19 (April – May 2020) (N=254 publications)	p-value
COVID-19-related post/episode			
No	868 (431-1657)* (n=546)†	1380 (537-3202) (n=184)	<0.0001
Yes	1520 (692-15494) (n=48)	4229 (965-15479) (n=70)	0.10
CME credit offered			
No	802 (340-3047) (n=183)	955 (420-2731) (n=158)	0.37
Yes	903 (523-1546) (n=411)	4039 (1870-11599) (n=96)	<0.0001
Journal affiliation			
No	879 (422-1768) (n=546)	2109 (624-4460) (n=207)	<0.0001
Yes	928 (740-2782) (n=48)	1101 (831-2769) (n=47)	0.30
Twitter followers			
<10k	951 (549-1782) (n=458)	2873 (1262-6784) (n=129)	<0.0001
>10k	516 (290-1968) (n=136)	852 (358-2248) (n=125)	0.05
Theme			
Clinical review	1025 (687-2311) (n=253)	1176 (707-3201) (n=106)	<0.0001
Narrative story	544 (277-945) (n=184)	2325 (1645-3945) (n=39)	<0.0001
Case-based discussion	1503 (1046-2849) (n=36)	2602 (1324-6745) (n=18)	0.06
Interview with investigator/author	1764 (1437-2165) (n=29)	2191 (802-12631) (n=14)	1.00
Other	651 (290-2450) (n=92)	795 (301-1975) (n=77)	0.89

In unadjusted linear regression, more downloads/pageviews were observed over the first 30 days following publication during COVID-19 than pre-COVID-19 (β=2.24, 95%CI 1.40-3.07, p<0.001). Over the study period as a whole, more 30-day downloads/pageviews were observed among podcasts versus blogs (β=2.48, 95%CI 1.52-3.44, p<0.001), among case-based discussions versus any other content theme (β=1.79, 95%CI 0.20-3.37, p=0.03), and among publications with fewer Twitter followers (β= -0.06, 95%CI -0.12-0.004, p=0.05). There was no association between 30-day downloads or pageviews and academic society or journal affiliation (p=0.22), nor the provision of CME credit (p=0.31). After adjusting for covariates associated with higher monthly downloads in univariate modeling (content theme of case-based discussion, presence of COVID-19 content, and Twitter followers), the final linear mixed model confirmed that publication during COVID-19 was associated with statistically higher 30-day downloads or pageviews (β=1.55, 95%CI 0.85-2.26, p<0.001). In this model, COVID-19 content (β=7.21, 95%CI 6.29-8.14, p<0.001) remained strongly associated with higher monthly downloads as did the theme of case-based discussion (β=1.49, 95%CI 0.24-2.74, p=0.02) while Twitter followers were no longer significant (p=0.78).

## Discussion

To our knowledge, this is the first study to systematically assess the change in blog and podcast use in medical education before and after the novel coronavirus created a global pandemic. This is important for the initial understanding of which parts of this medical education paradigm change will stay after the pandemic is behind us and may even help us understand possible “curricula shock” experienced by learners. Our study sampled a cross-section of FOAM educators and attempted to consider the heterogeneity of blog and podcast programs using a mixed-effects linear regression model and adjusting for differences between each program as well as differences between program types (blog vs. podcast). Our study found an increase in downloads or pageviews for new content published to FOAM platforms during the early COVID-19 pandemic, confirming our hypothesis of increased utilization. When broken down by media type, blogs showed a significant spike in access during the early pandemic while podcasts did not. The statistically significant increase in monthly pageviews was driven primarily by a single large blog (two-thirds of all posts analyzed). When this blog was removed from the analysis, the remaining blogs still had numerically increased pageviews, but this increase was no longer statistically significant (data not otherwise shown). COVID-19 content comprised more than one in 10 published blogs and podcasts in the first five months of 2020 (increasing to 1 in 4 after the pandemic declaration) and was clearly a strong predictor of higher downloads or pageviews.

These findings of increased utilization could be due to changing motivations to consume (or changing access to) blogs and podcasts for medical education and clinical learning during the pandemic, especially when related to COVID-19. Particularly when information is not readily available from traditional peer-reviewed resources, FOAM resources like blogs may offer an accessible medium for interpreting rapidly changing knowledge.

One surprising finding of this analysis is the lack of increase in podcast utilization during the early COVID-19 pandemic. This may be explained by podcasts differing from blogs as media forms. Unlike blogs, podcasts are infrequently accessed in isolation. By this, we mean podcast users are more likely to engage with episodes simultaneously with other activities (e.g., work commutes, exercise) while blogs require an individual’s full attention [13]. It is possible that early stay-at-home orders and restriction of students’ clinical rotations early during the pandemic may have created new opportunities for committed, self-directed learning using resources that require undivided attention (e.g., blogs). Conversely, the COVID-19 period may have given some learners more time such that multitasking was not any more necessary during this period. Podcasts are also more commonly curated by apps that may automatically download episodes. As such, even if listening to podcasts may have changed significantly, the collectable metric of "downloads" may not have changed. Listening to podcast episodes lasting 30-60 minutes also represents a greater time commitment than reading a blog post. Time pressures or attention fatigue, particularly in the early pandemic period, may also have led to a preference for blog utilization. As a point-of-care resource, blogs may have been more consumable in the healthcare workplace than podcasts. With increased interest and motivation to use FOAM, the need to ensure that these resources meet established criteria for quality will become ever more important. Several publications have already demonstrated the performance of evaluation metrics for use on these online resources [14-17].

Predictably, COVID-19 content was a strong predictor of higher utilization metrics, however, even non-COVID-19-related material was more frequently sought out during the early pandemic. We speculate that this demonstrates that learners and medical professionals were not just turning to FOAM resources for rapidly changing information about the pandemic but also as a resource for the full scope of medical education. Further study would need to be conducted to define the exact topics that are accessed more than others, though our study was at least able to show that “case-based discussions” were among the most frequently accessed style of programming.

Although traditional in-person didactic education will no doubt return after the pandemic, the quality, diversity, multimedia content, and convenience of available OERs will continue to attract learners and educators alike. As training programs begin incorporating OERs into traditional curricula in a blended approach, the use of a collaborative curation model may ensure quality content without overburdening busy educators [18]. Educators will need to view FOAM as a component for inclusion in their overall curriculum map, and thoughtfully consider the merit and role for these resources just as they do for the traditional educational elements they may replace or augment [19-21]. We believe that the first step in this possible lasting paradigm shift is to understand which content was more utilized and to what extent: a milestone we have made efforts to achieve with this paper.

Our findings also have important implications for the career development and advancement of educators. Until this point, many education scholars have found it necessary to write traditional papers about their online curricular projects to gain scholarly credit for their work, however, there is increasing interest to see these OERs as forms of digital scholarship that should be judged by their own merits [4]. As these asynchronous resources become more important within our learning environments, their development, peer review, and scholarly assessment of impact will increasingly need to be accepted as valued academic output for the purpose of faculty review and promotion. While a number of institutions have amended their criteria for promotion to include social media activity and non-traditional curriculum development, this is far from universal, and the specific standards applied are variable [11,22]. There has been recent work towards the development of consensus guidelines for digital scholarship for academic promotion [23]. While the work in this area may be preliminary, it forms a solid foundation upon which digital medical educators can grow their careers. With greater value placed on these resources, more educators may be afforded time and resources to develop and integrate asynchronous materials into their curricula. Over time, we are likely to see greater quantity (and quality) of these materials in medical education. 

Tracking blogpost and podcasting utilization on medical education carries a number of limitations. First and foremost, most metrics are related to downloads and distribution of digital content online. Although open distribution is a great strength of the online medium, this limits any ability for researchers to impose inclusion or exclusion criteria to monitor utilization among certain populations or strict outcome definitions across the heterogeneous platforms. No platform currently provides a way to directly monitor learner engagement with the content in a systematic and comprehensive fashion. Listener metrics for podcasts vary between download numbers, streaming, and content access and are often more closely related to digital content transfer rates from the distribution service to the reader or listener’s device than whether or not the learner consumed the content. In addition, the demographic data of listeners or blog viewers are also limited, and there is no current way to confirm whether users are actually trainees in the medical field. Therefore, extrapolating digital download numbers to medical trainee education and generalizing specifics of these data across platforms can be spurious. For example, we can imagine a layperson being more likely to view a medical blog post on COVID-19 than many other diseases, considering how pervasive the desire was for new information and pandemic literacy. The cut-off date for “pre-COVID-19” vs “early COVID-19” is also a limitation. It was not feasible to collect data that would accurately sort blog posts and podcasts based on the exact date their home country started stay-at-home orders or the virtual changes to medical education. Considering the inconsistent implementation of lockdowns throughout March, April was selected as a reasonable starting month for stay-at-home education. These data are exploratory and were collected openly but selectively over social media in the spirit of online connectivity and did not adjust for multiple hypothesis testing. For this reason, these data should be used for hypothesis generation rather than definitive conclusions about the utilization of podcasting in medical education.

## Conclusions

In a shifting landscape for medical education, Free Open Access Medical education may play an important role in rapid knowledge translation. Our study showed increased use of blogs during the initial months of the COVID-19 pandemic but no increase in podcast use. Future research should evaluate the impact of blogs and podcasts compared to other forms of virtual and traditional modes of education.
